# Characterizing HIV epidemiology in stable couples in Cambodia, the Dominican Republic, Haiti, and India

**DOI:** 10.1017/S0950268815000758

**Published:** 2015-04-28

**Authors:** H. CHEMAITELLY, L. J. ABU-RADDAD

**Affiliations:** 1 Infectious Disease Epidemiology Group, Weill Cornell Medical College – Qatar, Cornell University, Qatar Foundation – Education City, Doha, Qatar; 2 Department of Healthcare Policy and Research, Weill Cornell Medical College, Cornell University, NY, USA; 3 Vaccine and Infectious Disease Division, Fred Hutchinson Cancer Research Center, Seattle, WA, USA

**Keywords:** Demographic and Health Surveys, epidemiology, HIV incidence, serodiscordancy, stable couples, transmission

## Abstract

Using a set of statistical methods and HIV mathematical models applied on nationally representative Demographic and Health Survey data, we characterized HIV serodiscordancy patterns and HIV transmission dynamics in stable couples (SCs) in four countries: Cambodia, the Dominican Republic, Haiti, and India. The majority of SCs affected by HIV were serodiscordant, and about a third of HIV-infected persons had uninfected partners. Overall, nearly two-thirds of HIV infections occurred in individuals in SCs, but only about half of these infections were due to transmissions within serodiscordant couples. The majority of HIV incidence in the population occurred through extra-partner encounters in SCs. There is similarity in HIV epidemiology in SCs between these countries and countries in sub-Saharan Africa, despite the difference in scale of epidemics. It appears that HIV epidemiology in SCs may share similar patterns globally, possibly because it is a natural ‘spillover’ effect of HIV dynamics in high-risk populations.

Stable couples (SCs) have become a priority for HIV prevention efforts following the demonstrated efficacy of several prevention interventions among them in averting HIV heterosexual transmission [1, 2]. Characterizing HIV epidemiology in SCs has become critical for informing HIV policy and programming [2].

We recently conducted a quantitative assessment of HIV epidemiology in SCs in sub-Saharan Africa (SSA). We provided a mapping of HIV serodiscordancy patterns [3] and assessed the sources of HIV infection in SCs, including stable HIV serodiscordant couples (SDCs), and their contribution to HIV population-level incidence [4–6]. While HIV epidemiology in SCs has been researched in SSA through numerous studies, little is known about the patterns of HIV serodiscordancy and HIV transmission dynamics in SCs outside the African context. We extend here our earlier analyses to all countries outside SSA for which HIV biomarker Demographic and Health Survey (DHS) data are available.

We analysed the most recent DHS surveys for Cambodia (2005), the Dominican Republic (2007) including a sub-population characterized by higher HIV prevalence (Bateyes-Dominican Republic, 2007), Haiti (2012), and India (2005–2006). Vietnam was excluded from our analysis because of the low number of couples affected by HIV (only three couples), which hindered meaningful analysis.

Following DHS methodology, we defined a SC as a man and a woman living in a consensual union within a household at the time of the survey [7]. SCs where one or both partners did not test for HIV were excluded from our analysis. Missing HIV information in all SCs was measured at 2·1% in Cambodia, 5·7% in the Dominican Republic, 3·5% in Bateyes-Dominican Republic, 3·4% in Haiti, and 3·6% in India.

We characterized HIV serodiscordancy patterns by quantifying the proportion of SDCs in all SCs in the population, the proportion of SDCs in all SCs with at least one HIV-infected individual in the couple, the prevalence of couples affected by HIV in all SCs, the proportion of individuals who are engaged in a SDC in the entire population of reproductive age, and the proportion of HIV-infected individuals engaged in SDCs. The methodology for the derivation of these measures can be found in the Supplementary material and in Chemaitelly *et al.* [3].

We described HIV transmission dynamics in SCs using a set of established mathematical models for characterizing HIV epidemiology in SCs. Details on these models, their parameterization, measures' derivations, and analysis methodology can be found in the Supplementary material and in our earlier publications [4–6]. Briefly, we quantified the fraction of new HIV infections arising every year in SDCs due to extra-couple HIV exposure of the uninfected partner. We also quantified the contribution of SCs to total HIV incidence in the population stratified by couples' status and source of HIV infection. Specifically, we assessed the contribution of stable concordant HIV-negative couples to HIV population-level incidence due to (*a*) external HIV acquisition by one of the partners in the couple, (*b*) external HIV acquisition by both partners in the couple, and (*c*) HIV transmission to the uninfected partner shortly after the external acquisition of HIV by the other partner (index partner). We also assessed the contribution of SDCs to HIV population-level incidence due to (*a*) HIV transmission from the infected to the uninfected partner in the couple, and (*b*) the external acquisition of HIV by the uninfected partner in the couple.

Uncertainty analyses were performed for each of the modelled country-specific measures by implementing 10 000 runs of the models using Monte Carlo sampling from triangular probability distributions for the confidence intervals (CIs) or plausibility ranges of the demographic, biological, and epidemiological parameters. Country-specific distributions for these measures were then generated and used to calculate the mean and associated 95% CIs.

The socio-demographic and health characteristics of the male, female and SC populations across countries can be found in Supplementary Tables S1 and S2. Overall, the countries are characterized by low HIV prevalence, measured at <1% in India, Cambodia, and the Dominican Republic, at 2·2% in Haiti and at 3·3% in Bateyes-Dominican Republic. The majority of the population of reproductive age in these countries is engaged in SCs (Fig. 1*a*). A substantial fraction of HIV-infected individuals are also in SCs: 69% in India, 76% in Cambodia, 56% in the Dominican Republic, 60% in Haiti, and 65% in Bateyes-Dominican Republic (Fig. 1*b*). If we were to randomly draw couples from the population, less than one couple in every 100 couples would be HIV serodiscordant in each of India, Cambodia, and the Dominican Republic, compared to three couples in Haiti and four couples in Bateyes-Dominican Republic (Fig. 1*c*). Moreover, less than one couple in every 100 couples would be affected by HIV (serodiscordant or concordant positive) in India, compared to one couple in Cambodia and the Dominican Republic, four couples in Haiti and six couples in Bateyes-Dominican Republic.

**Fig. 1.**
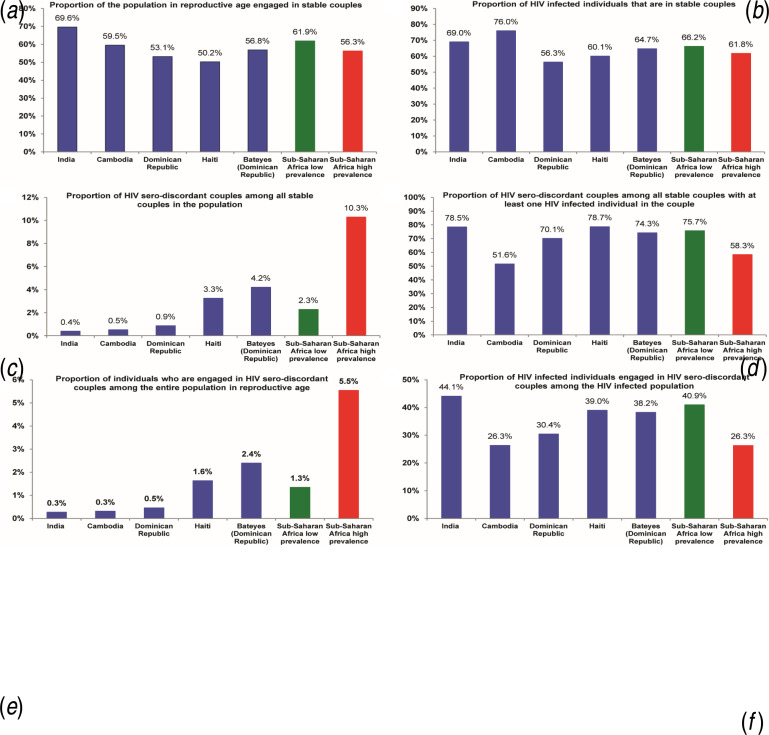
Patterns of HIV serodiscordancy in India, Cambodia, the Dominican Republic, and Haiti compared to those in low and high HIV prevalence countries in sub-Saharan Africa. Countries are shown in order of increasing HIV prevalence.

In couples affected by HIV, 78% were serodiscordant in India, 52% in Cambodia, 70% in the Dominican Republic, 79% in Haiti, and 74% in Bateyes-Dominican Republic (Fig. 1*d*). While a small fraction of individuals of reproductive age are engaged in a SDC (less than one out of every 100 individuals in India, Cambodia, and the Dominican Republic, *vs.* two in each of Haiti and Bateyes-Dominican Republic; Fig. 1*e*), a large fraction of HIV-infected individuals have uninfected partners (44% in India, 26% in Cambodia, 30% in the Dominican Republic, 39% in Haiti, and 38% in Bateyes-Dominican Republic; Fig. 1*f*).

Most HIV incidence in SDCs is due to HIV transmission from the infected to the uninfected partner within the couple (Fig. 2*a*). Our findings also show that males constitute the majority of index partners in SDCs in India (82·0%) and in Cambodia (83·4%). Meanwhile, the distribution of index partners between males and females is more or less equal in the Dominican Republic (51·1% males), Haiti (43·5% males) and Bateyes-Dominican Republic (49·3% males).

**Fig. 2.**
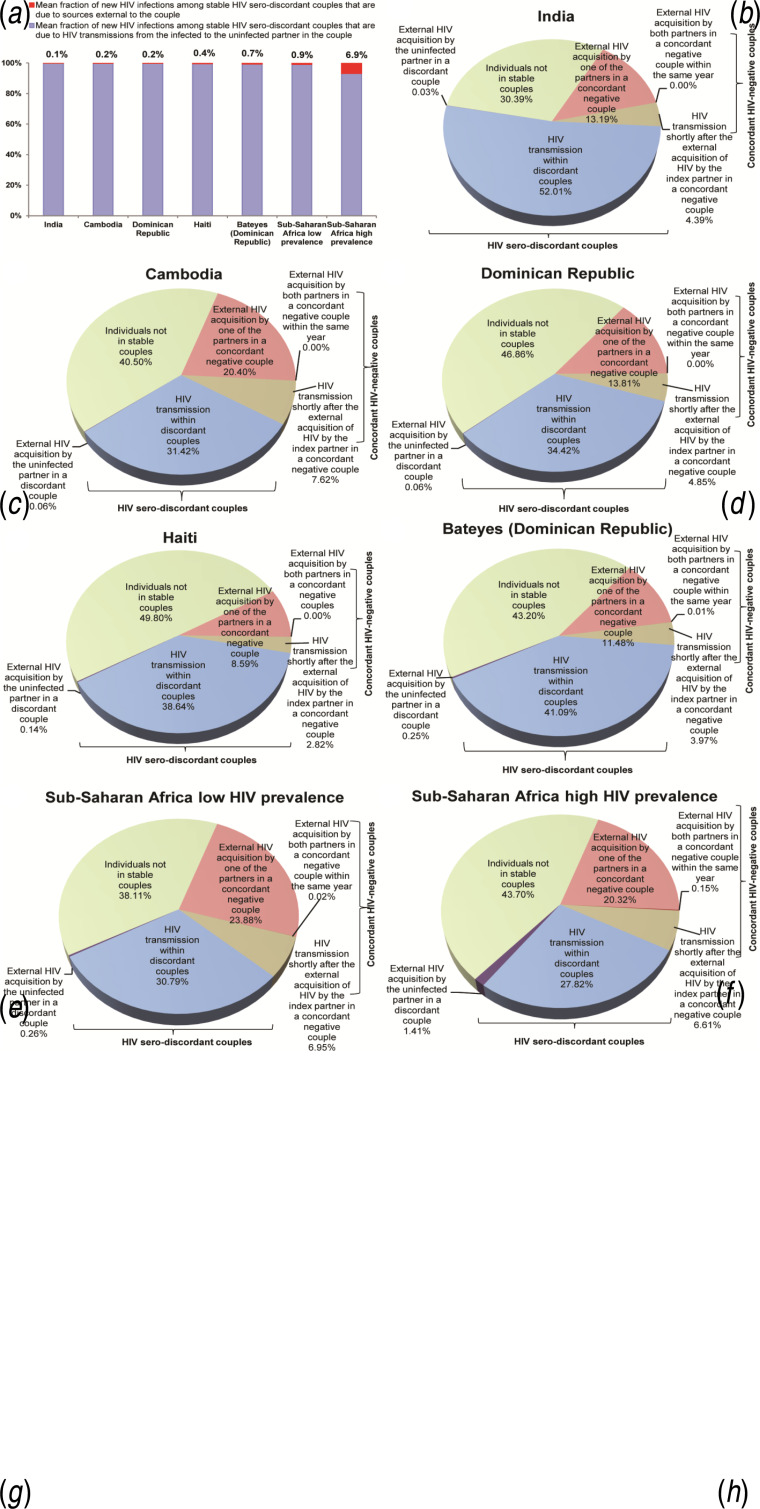
HIV incidence and its sources in stable couples. (*a*) Fraction of new HIV infections in stable HIV serodiscordant couples that are due to sources external *vs.* internal to the couple. Estimates are shown for HIV incidence arising in stable HIV serodiscordant couples due to sources external to the couple. (*b*–*h*) The mean country-specific contributions to HIV incidence in the population stratified by couples' serostatus and source of HIV infection. Results are displayed for India, Cambodia, the Dominican Republic, and Haiti, compared to those in low and high HIV prevalence countries in sub-Saharan African. Countries are shown in order of increasing HIV prevalence.

HIV incidence in SDCs due to external sources contributes <0·3% of HIV population-level incidence in these countries (Fig. 2*b*–*f*). HIV transmissions from the infected to the uninfected partner in SDCs contributes 52·0% (95% CI 31·9–68·8) of HIV population-level incidence in India, 31·4% (95% CI 16·4–51·0) in Cambodia, 34·4% (95% CI 20·3–50·0) in the Dominican Republic, 38·6% (95% CI 24·1–50·3) in Haiti, and 41·1% (95% CI 23·2–57·4) in Bateyes-Dominican Republic (Fig. 2*b–f*).

In stable concordant HIV-negative couples, HIV incidence due to one partner acquiring the infection from a source external to the couple contributes 13·2% (95% CI 1·0–30·0) of HIV population-level incidence in India, 20·4% (95% CI 5·8–33·6) in Cambodia, 13·8% (95% CI 2·5–26·0) in the Dominican Republic, 8·6% (95% CI 0·5–20·6) in Haiti, and 11·5% (95% CI 0·8–25·5) in Bateyes-Dominican Republic (Fig. 2*b–f*). Only 4·4% (95% CI 0·4–8·2) of new HIV infections in India, 7·7% (95% CI 2·7–11·2) in Cambodia, 4·9% (95% CI 1·1–7·5) in the Dominican Republic, 2·8% (95% CI 0·2–5·6) in Haiti, and 4·0% (95% CI 0·3–7·7) in Bateyes-Dominican Republic, are due to HIV transmission from the infected to the uninfected partner in the couple within less than a year after the infected partner had acquired HIV from an external source (Fig. 2*b–f*). The virtually vanishing fraction of HIV population-level incidence is due to both partners acquiring HIV from an external source within the same year (Fig. 2*b*–*f*).

The remaining HIV population-level incidence occurs in persons not in a SC (Fig. 2*b–f*): 30·4% (95% CI 26·3–34·5) in India, 40·5% (95% CI 40·2–40·9) in Cambodia, 46·9% (95% CI 44·1–49·6) in the Dominican Republic, 49·8% (95% CI 46·3–53·3) in Haiti, and 43·2% (95% CI 36·4–50·1) in Bateyes-Dominican Republic.

We described the patterns of HIV serodiscordancy and examined HIV transmission dynamics in SCs in Cambodia, the Dominican Republic, Haiti, and India. We found that just over half of the population of reproductive age is engaged in SCs in these countries (Fig. 1*a*). Although a small minority of SCs is affected by HIV (Fig. 1*c*), the majority of the couples affected by HIV are serodiscordant (Fig. 1*d*). About two-thirds of HIV-infected individuals are engaged in SCs (Fig. 1*b*), and one-third have uninfected partners (Fig. 1*f*).

HIV incidence in the population is distributed roughly equally in SDCs, concordant HIV-negative couples, and individuals not in a SC (Fig. 2*b–f*). In SDCs, most new HIV infections occur when the infected partner transmits the infection to the uninfected partner. Rarely do uninfected individuals in SDCs acquire the infection externally, even if they engage in extra-marital sex. The small risk of forming an extra-marital partnership with an infected person, with the low HIV prevalence, makes this mode of exposure insignificant compared to the risk of acquiring the infection from the infected partner in the SDC.

Nonetheless, in concordant HIV-negative couples, most HIV infections (about 75%) occur when one of the partners acquires the infection through extra-marital sex and becomes the index partner in the couple, and about a quarter of infections occur when a partner, who acquired the infection externally, passes the infection to the other partner shortly after acquiring the infection. With the low HIV prevalence in these countries, the contribution to HIV incidence of both partners acquiring the infection externally, within the same year, is virtually vanishing. Overall, of all HIV incidence in the population, about 20% arise by the creation of an index partner in initially concordant HIV-negative couples.

Although the main features of HIV epidemiology in SCs are similar across these countries, there are quantitative differences across the modes of exposure. These differences arise because of differences in engagement in SCs, HIV prevalence and incidence, level of serodiscordancy, coverage of male circumcision, and condom use, among others. For example, in India, SDCs were predicted to contribute about half of HIV incidence, larger than the contribution in the other countries. This outcome is a consequence of higher engagement in SCs (70%), higher level of serodiscordancy in partnerships affected by HIV (79%), lower coverage of male circumcision (13%), and lower level of condom use (6%).

HIV serodiscordancy patterns and transmission dynamics in SCs observed in these countries are overall similar to those characterized recently in low HIV prevalence countries in SSA (Figs 1 and 2) [3–6]. Indeed, several features are also similar to those seen in high HIV prevalence countries in SSA (Figs 1 and 2) [3–6]. It appears that there is some kind of ‘universality’ in HIV transmission dynamics in SCs, especially so in countries with low HIV prevalence (<5%). This universality may stem from the strong disposition of human populations to form stable marital partnerships, while at the same time, having a tendency towards engagement in extra-marital sex [8].

It is striking that such universality appears to exist even though the core drivers of HIV epidemics may vary from one country to another, such as the role of commercial sex *vs.* men who have sex with men or people who inject drugs. The universality probably arises because HIV epidemiology in heterosexual SCs in most countries appears to be simply a predictable ‘spillover’ effect of the ‘core’ HIV dynamics in high-risk populations. This conjecture, however, cannot be validated without first exploring HIV dynamics in mathematical models that incorporate both stable couples and high-risk groups, such as female sex workers and their clients, and track onward transmission in the population. It should be noted that the high fraction of male index partners in SDCs in India and Cambodia may suggest an important role for commercial sex in the HIV epidemics in these countries.

In light of these findings, a multi-component prevention approach addressing the various sources of HIV incidence in the population is required to substantially reduce HIV transmission in these countries. Increasing access to antiretroviral therapy (ART) among other prevention interventions for individuals at high risk of infection, such as within commercial sex networks, could produce a considerable decline in HIV incidence. Meanwhile, high ART coverage in SDCs can prevent most within-couple transmission [9]. Identifying SDCs in low HIV prevalence and resource-limited countries, however, could be challenged by logistical difficulties that may undermine the cost-effectiveness of the intervention.

Our analysis is limited by the availability of DHS HIV surveys only in specific countries, and the smaller samples of infected individuals in these countries compared to SSA. Some measures may have been biased due to inherent biases in DHS data, such as variability in response rates and selection bias in considering only couples with complete HIV serostatus information. The modelling estimates may have been affected by data inconsistencies resulting from the use of multiple data sources that follow different methodologies. HIV transmission probability per coital act and coital frequency may vary across countries [10], but we assumed equal levels in absence of country-specific data. To accommodate uncertainties, we chose a conservative approach of wider parameter uncertainty ranges and incorporation of uncertainty in all model parameters, leading to large CIs.

We estimated the contribution of SCs to HIV population-level incidence arising over the course of one year using cohort-type models defined on a cohort of SCs according to the DHS cross-sectional surveys. Accordingly, our model does not incorporate dynamical partnership formation and dissolution. This limitation, however, is unlikely to affect our results given the long marital durations indicated by the analysis of DHS data (Supplementary Table S2), and the fact that there should be roughly an equilibrium between new partnership formation and dissolution in the population. Moreover, HIV incidence in SCs that is regarded as onward transmission of index infections acquired in the past, is effectively included in our estimates since these specific SCs define the SDC populations in the modelled cohort.

The type of our model along with the cross-sectional nature of DHS data, where the history of infected individuals cannot be tracked, limit our ability to determine whether index partners in SDCs acquired the infection during or prior to engaging in a SC, or whether infected individuals not in a SC acquired the infection in a dissolved partnership.

In conclusion, there is similarity in HIV patterns in SCs between these countries and SSA countries, suggesting that HIV epidemiology in SCs may be a predictable ‘spillover’ effect of HIV dynamics in high-risk populations. HIV incidence in individuals in SCs is a large fraction of all HIV incidence in any population, but only part of this incidence is occurring when an infected partner passes the infection to the uninfected partner within the couple. Intervention programmes should consider the patterns of HIV serodiscordancy and modes of HIV exposure in SCs as part of programme design to ensure optimal roll-out of interventions.

## Supplementary material

For supplementary material accompanying this paper visit http://doi.org/10.1017/S0950268815000758. click here to view supplementary material
