# Supplementary material for: Characterizing HIV epidemiology in stable couples in Cambodia, the Dominican Republic, Haiti, and India
Source: Epidemiol Infect. 2015 Apr 28;144(1):90–6. doi: 10.1017/S0950268815000758 (PMC4697301; doi:10.1017/S0950268815000758)
Supplement: Supplementary file 1 [file S0950268815000758sup001.docx]

***Epidemiology and Infection***

**Characterizing HIV epidemiology among stable couples in Cambodia, the Dominican Republic, Haiti, and India**

H. Chemaitelly^1*^ and L.J. Abu-Raddad^1,2^,^3^

**Supplementary Material**

^1^Infectious Disease Epidemiology Group, Weill Cornell Medical College - Qatar, Cornell University, Qatar Foundation - Education City, Doha, Qatar

^2^Department of Healthcare Policy and Research, Weill Cornell Medical College, Cornell University, New York, USA

^3^Vaccine and Infectious Disease Division, Fred Hutchinson Cancer Research Center, Seattle, Washington, USA

Reprints or correspondence: Hiam Chemaitelly, MSc, Weill Cornell Medical College – Qatar, Qatar Foundation - Education City, P.O. Box 24144, Doha, Qatar. Telephone: +(974) 4492-8443. Fax: +(974) 4492-8422. E-mail: hsc2001@qatar-med.cornell.edu.

**Further methodological details describing the data analysis process**

We include below a summary of key aspects of the methodologies used in this study. Detailed descriptions of these methodologies, including statistical methods and mathematical models and their parameterization, can be found in earlier publications [[1-4](#_ENREF_1)].

1. ***Characterizing HIV sero-discordancy patterns***

We defined several epidemiological HIV sero-discordancy measures (Figure 1C-1F):

1. The proportion of HIV sero-discordant couples (SDCs) among all stable couples (SCs) in the population () defined as:

This measure conveys the level of discordancy among all SCs in the population (Figure 1C).

1. The proportion of SDCs among all SCs with at least one HIV infected individual in the couple () defined as:

This measure conveys the proportion of SCs affected by HIV where the uninfected partner has not acquired the infection yet, but is at risk of acquiring it from the infected partner in the future (Figure 1D).

1. The proportion of individuals who are engaged in SDCs among the entire population in reproductive age () defined as:

This measure conveys the abundance of individuals who are engaged in SDCs in the population (Figure 1E).

1. The proportion of HIV infected individuals engaged in SDCs among the HIV infected population () defined as:

This measure conveys the level of engagement of HIV infected individuals in SDCs (Figure 1F).

These measures were derived using calculated country-specific demographic and epidemiological indicators available from the Demographic and Health Surveys (DHS) [[5](#_ENREF_5)] and the World Population Prospects database [[6](#_ENREF_6)]. These include the size of the population in reproductive age, the proportion of the population in reproductive age engaged in SCs (using the average rate of self-reported engagement in SCs for males and females), the proportion of HIV infected individuals engaged in SCs (using the average rate of self-reported engagement in SCs for HIV infected males and females), the distribution of partnerships based on HIV sero-status (concordant HIV-negative, HIV sero-discordant, or concordant HIV-positive), the number of HIV infected individuals, and HIV population prevalence. Further details pertaining to these measures and their derivations can be found in an earlier publication [[4](#_ENREF_4)].

1. ***Contribution of HIV incidence among SCs to HIV population-level incidence***

We used a cohort-type mathematical model to calculate, for each country, the contribution of six different types of HIV incidence, stratified by couple status and source of infection, to HIV incidence arising in the population over the course of a given year of observation. The six HIV incidence measures include:

Among stable concordant HIV-negative couples,

1. The external HIV acquisition by one of the partners in the couple measured using the expression $2\lambda\left(1-\lambda\right)N_{couples}P_{SCNC}$. Here, $\lambda$ is the probability of an HIV sero-negative partner in a SC to acquire the infection from a source external to the couple over the course of one year (as defined in the previous section above). $N_{couples}$ is the number of SCs estimated through the baseline screening cross-sectional survey, and $P_{SCNC}$ is the prevalence of stable concordant HIV-negative couples among all couples.
2. The external HIV acquisition by both partners in the couple measured using the expression $2\lambda^{2}N_{couples}P_{SCNC}$.
3. HIV transmission to the uninfected partner shortly after the external acquisition of HIV by the index partner measured using the expression $2t_{6mths}\lambda N_{couples}P_{SCNC}$. In this expression, $t_{6mths}$ is the probability that the index partner who acquired the infection from an external source will transmit the infection to the uninfected partner during the six months following the acquisition of HIV. Here we assume that the index partners acquired the infection externally, on average, in the middle of the year of observation.

Among SDCs,

1. HIV transmission from the infected to the uninfected partner in the couple measured using the expression $t_{1year}N_{couples}P_{all}$. Here, $t_{1year}$ is the probability that the index partner in an SDC will transmit the infection to the uninfected partner over the course of the year of observation.
2. The external acquisition of HIV by the uninfected partner in the couple measured using the expression $\lambda N_{couples}P_{all}$.

Among susceptible individuals not in SCs, HIV incidence among these individuals is estimated using the expression . Here, $N_{rep\_age}$ is the size of the population in reproductive age, $f_{in\_couples}$ is the fraction of the population in reproductive age engaged in SCs, is HIV population prevalence, and $\theta$ is HIV population-level incidence rate. $\theta$ is derived from DHS HIV population prevalence using the expression: $\theta=\frac{HIV population pevalence}{Duration of infection}$ [[7](#_ENREF_7)].

HIV population-level incidence is defined as the number of new HIV infections arising within a year among susceptible individuals in reproductive age. This measure defines the denominator in all six contribution measures, and is calculated using $\theta$ and the estimated number of uninfected individuals in reproductive age for each country.

The likelihood of an HIV sero-negative partner in a SC to acquire the infection from a source external to the SC (that is $\lambda$) was determined by the condition that HIV population-level incidence, as estimated from pooling together all incidence measures among SCs and individuals not in SCs, must be equal to the total HIV incidence estimated independently using $\theta$ and the number of uninfected individuals in reproductive age for each country. All incidence measures were then recalculated using this fitted value of $\lambda$, and estimates for the contributions of new HIV infections among SCs to total HIV incidence in the population were derived.

The fraction of HIV incidence arising among SDCs due to acquiring the infection from a source external to the couple by the uninfected partner ($f_{ext}$) was determined by comparing HIV incidence among SDCs due a source external to the SDC to total HIV incidence among SDCs.

The models were parameterized using epidemiological indicators from the DHS [[5](#_ENREF_5)] and state-of-art data on HIV transmission and HIV natural history [[8-10](#_ENREF_8)].

**Table S1.** Characteristics of the male and female populations in India, Cambodia, the Dominican Republic, Haiti, and Bateyes-Dominican Republic.

| **Population characteristics** | **India** | | **Cambodia** | | **Dominican Republic** | | **Haiti** | | **Bateyes-Dominican Republic** | |
| --- | --- | --- | --- | --- | --- | --- | --- | --- | --- | --- |
|  | **Males** | **Females** | **Males** | **Females** | **Males** | **Females** | **Males** | **Females** | **Males** | **Females** |
| **Age** |  |  |  |  |  |  |  |  |  |  |
| Mean ± SE | 31.1 ± 0.05 | 29.0 ± 0.04 | 28.8 ± 0.15 | 29.8 ± 0.10 | 32.2 ± 0.11 | 29.7 ± 0.09 | 30.9 ± 0.15 | 28.2 ± 0.10 | 32.6 ± 0.32 | 28.8 ± 0.26 |
| **Place of residence** (%) |  |  |  |  |  |  |  |  |  |  |
| Urban | 36.51 | 32.82 | 16.84 | 17.67 | 68.76 | 71.83 | 44.26 | 47.50 |  |  |
| Rural | 63.49 | 67.18 | 83.16 | 82.33 | 31.24 | 28.17 | 55.74 | 52.50 | 100 | 100 |
| **Educational attainment** (%) | | | | | | | | | | |
| No education | 18.53 | 40.59 | 9.00 | 19.44 | 4.16 | 3.39 | 13.03 | 14.80 | 19.03 | 16.08 |
| Primary education | 17.02 | 14.70 | 48.44 | 55.81 | 46.09 | 37.51 | 36.99 | 36.27 | 58.63 | 57.73 |
| Secondary education | 52.02 | 37.41 | 39.21 | 23.63 | 35.63 | 38.97 | 42.32 | 43.32 | 19.86 | 23.28 |
| Higher education | 12.42 | 7.30 | 3.35 | 1.13 | 14.12 | 20.13 | 7.65 | 5.60 | 2.49 | 2.91 |
| **Wealth index** (%) |  |  |  |  |  |  |  |  |  |  |
| Poorest | 15.78 | 17.46 | 16.01 | 17.93 | 21.38 | 15.67 | 17.93 | 15.22 | 47.66 | 34.44 |
| Poorer | 18.08 | 18.99 | 18.10 | 18.81 | 19.86 | 19.22 | 18.21 | 16.17 | 28.48 | 34.75 |
| Middle | 20.39 | 20.17 | 20.08 | 19.29 | 19.77 | 21.04 | 19.75 | 19.66 | 15.54 | 19.15 |
| Richer | 22.16 | 20.99 | 21.81 | 19.66 | 19.87 | 21.70 | 21.25 | 22.81 | 6.78 | 9.11 |
| Richest | 23.59 | 22.40 | 24.00 | 24.30 | 19.13 | 22.37 | 22.87 | 26.12 | 1.53 | 2.55 |
| **Marital status** (%) |  |  |  |  |  |  |  |  |  |  |
| Never married | 34.13 | 20.47 | 38.71 | 31.81 | 36.59 | 24.00 | 48.53 | 36.94 | 34.13 | 18.76 |
| Currently married | 64.37 | 74.84 | 59.03 | 59.96 | 49.58 | 56.69 | 45.72 | 54.65 | 47.98 | 65.7 |
| Formerly married | 1.50 | 4.69 | 2.26 | 8.23 | 13.83 | 19.31 | 5.75 | 8.41 | 17.89 | 15.54 |
| **Condom use at last intercourse** (%) | | | | | | | | | | |
| No | 91.27 | 94.28 | 88.72 | 97.09 | 69.75 | 88.53 | 63.55 | 78.77 | 71.58 | 90.74 |
| Yes | 8.73 | 5.72 | 11.28 | 2.91 | 30.25 | 11.47 | 36.45 | 21.23 | 28.42 | 9.26 |
| **Male circumcision** (%) |  |  |  |  |  |  |  |  |  |  |
| No | 87.03 |  | 97.9 |  | 85.33 |  | 94.17^*^ |  | 91.71 |  |
| Yes | 12.97 |  | 2.1 |  | 14.67 |  | 5.83^*^ |  | 8.29 |  |
| **HIV infected** (%) |  |  |  |  |  |  |  |  |  |  |
| No | 99.65 | 99.78 | 99.37 | 99.39 | 99.21 | 99.25 | 98.21 | 97.33 | 96.48 | 96.89 |
| Yes | 0.35 | 0.22 | 0.63 | 0.61 | 0.79 | 0.75 | 1.79 | 2.67 | 3.52 | 3.11 |
| **Total N** | **74 369** | **124 385** | **6 731** | **16 823** | **27 975** | **27 195** | **9 493** | **14 287** | **1 820** | **1 575** |

^*^Information on male circumcision was not available in the 2012 Demographic and Health Survey for Haiti. The reported information is extracted from a previous survey conducted in 2005.

**Table S2.** Stable couples’ and other characteristics in India, Cambodia, the Dominican Republic, Haiti, and Bateyes-Dominican Republic.

| **Characteristics** | **India** | **Cambodia** | **Dominican Republic** | **Haiti** | **Bateyes-Dominican Republic** |
| --- | --- | --- | --- | --- | --- |
| Population (N) | 1 127 144 000 | 13 356 000 | 9 343 000 | 10 250 200 | 98 540 |
| Proportion of population in reproductive age (%) | 51.05 | 47.80 | 53.70 | 51.90 | 44.65 |
| Proportion of population in reproductive age in SCs (%) | 69.61 | 59.50 | 53.14 | 50.20 | 56.84 |
| Sample size of SCs (n) | 39 257 | 3 732 | 10 847 | 3 085 | 676 |
| Proportion of males in SCs (%) | 64.37 | 59.03 | 49.58 | 45.73 | 47.98 |
| Proportion of females in SCs (%) | 74.84 | 59.96 | 56.69 | 54.66 | 65.70 |
| Marital duration (mean in years) | 13.67 | 12.75 | 14.18 | 11.98 | 13.90 |
| Number of couples that tested for HIV (n) | 26 230 | 3 564 | 9 732 | 2 943 | 639 |
| HIV population prevalence (%) | 0.28 | 0.62 | 0.77 | 2.23 | 3.33 |
| Prevalence of SDCs among SCs (%) | 0.39 | 0.52 | 0.87 | 3.25 | 4.21 |
| Prevalence of positive concordancy among SCs (%) | 0.11 | 0.49 | 0.37 | 0.88 | 1.46 |
| Prevalence of negative concordancy among SCs (%) | 99.51 | 98.99 | 98.76 | 95.88 | 94.33 |
| Proportion of male index partners among SDCs (%) | 82.02 | 83.36 | 51.12 | 43.46 | 49.34 |
| Condom use at last intercourse among SCs (%) | 5.69 | 2.82 | 3.29 | 7.96 | 3.13 |
| Proportion of male circumcision among males in SCs (%) | 12.69 | 2.29 | 16.39 | 6.37^**^ | 7.94 |
| Proportion of SDCs where male is circumcised if female is HIV infected (%) | 6.45 | 0 | 7.51 | 0^**^ | 0 |
| Age among SCs |  |  |  |  |  |
| Age of males (mean ± SE) | 36.7 ± 0.05 | 34.9 ± 0.15 | 37.7 ± 0.14 | 38.8 ± 0.27 | 36.9 ± 0.39 |
| Age of females (mean ± SE) | 31.3 ± 0.05 | 23.8 ± 0.16 | 32.7 ± 0.13 | 33.8 ± 0.27 | 30.9 ± 0.35 |
| Wealth index among SCs (%) |  |  |  |  |  |
| Poorest | 16.83 | 17.93 | 19.36 | 21.51 | 36.93 |
| Poorer | 19.61 | 19.71 | 22.28 | 21.58 | 34.75 |
| Middle | 21.55 | 20.91 | 21.49 | 19.22 | 17.83 |
| Richer | 21.26 | 20.91 | 19.28 | 19.67 | 8.35 |
| Richest | 20.75 | 20.54 | 17.59 | 18.01 | 2.14 |
| Educational attainment among SCs (%) |  |  |  |  |  |
| Educational attainment among males in SCs |  |  |  |  |  |
| No education | 23.73 | 12.00 | 4.75 | 28.0 | 18.12 |
| Primary education | 20.33 | 52.07 | 49.94 | 41.65 | 58.75 |
| Secondary education | 44.62 | 33.16 | 30.53 | 26.2 | 19.92 |
| Higher education | 11.32 | 2.77 | 14.78 | 4.16 | 3.21 |
| Educational attainment among females in SCs |  |  |  |  |  |
| No education | 46.18 | 22.59 | 4.61 | 41.52 | 18.92 |
| Primary education | 15.30 | 58.07 | 45.61 | 35.21 | 60.37 |
| Secondary education | 32.51 | 18.74 | 32.64 | 20.60 | 17.73 |
| Higher education | 6.01 | 0.61 | 17.14 | 2.67 | 2.99 |
| Place of residence among SCs (%) |  |  |  |  |  |
| Urban | 33.56 | 14.22 | 66.89 | 36.71 |  |
| Rural | 66.44 | 85.78 | 33.11 | 63.29 | 100 |

^*^SCs: stable couples; SDCs: HIV sero-discordant couples

^**^Information on male circumcision was not available in the 2012 Demographic and Health Survey for Haiti. The reported information is extracted from a previous survey conducted in 2005.

**REFERENCES**

(1) **Chemaitelly H, et al.** Sources of HIV incidence among stable couples in sub-Saharan Africa. *Journal of the International AIDS Society* 2014; **17**: 18765.

(2) **Chemaitelly H, et al.** Only a fraction of new HIV infections occur within identifiable stable discordant couples in sub-Saharan Africa. *Aids* 2013; **27**(2): 251-260.

(3) **Chemaitelly H, Abu-Raddad LJ.** External infections contribute minimally to HIV incidence among HIV sero-discordant couples in sub-Saharan Africa. *Sexually Transmitted Infections* 2013; **89**(2): 138-141.

(4) **Chemaitelly H, et al.** Distinct HIV discordancy patterns by epidemic size in stable sexual partnerships in sub-Saharan Africa. *Sexually Transmitted Infections* 2012; **88**(1): 51-57.

(5) **MEASURE DHS**. Demographic and health surveys. Calverton: ICF Macro; 2012.

(6) **World Population Prospects.** (<http://esa.un.org/unpd/wpp/unpp/panel_population.htm)>. Accessed January 2014.

(7) **Nelson KE, Williams CM**. *Infectious disease epidemiology : theory and practice*. 2nd ed. Sudbury, Mass.: Jones and Bartlett Publishers, 2007: pp. xvi, 1207 p.

(8) **Hughes JP, et al.** Determinants of Per-Coital-Act HIV-1 Infectivity Among African HIV-1-Serodiscordant Couples. *Journal of Infectious Diseases* 2012; **205**(3): 358-365.

(9) **Wawer MJ, et al.** Rates of HIV-1 transmission per coital act, by stage of HIV-1 infection, in Rakai, Uganda. *Journal of Infectious Diseases* 2005; **191**(9): 1403-1409.

(10) **Celum C, et al.** Acyclovir and transmission of HIV-1 from persons infected with HIV-1 and HSV-2. *New England Journal of Medicine* 2010; **362**(5): 427-439.
